# Supplementary material for: The causal relationship between the human gut microbiota and pyogenic arthritis: a Mendelian randomization study
Source: Front Cell Infect Microbiol. 2024 Nov 26;14:1452480. doi: 10.3389/fcimb.2024.1452480 (PMC11629706; doi:10.3389/fcimb.2024.1452480)
Supplement: Supplementary file 1 [file DataSheet1.zip › mendelian test/ebi-a-GCST90027801.csv_funnel.pdf]

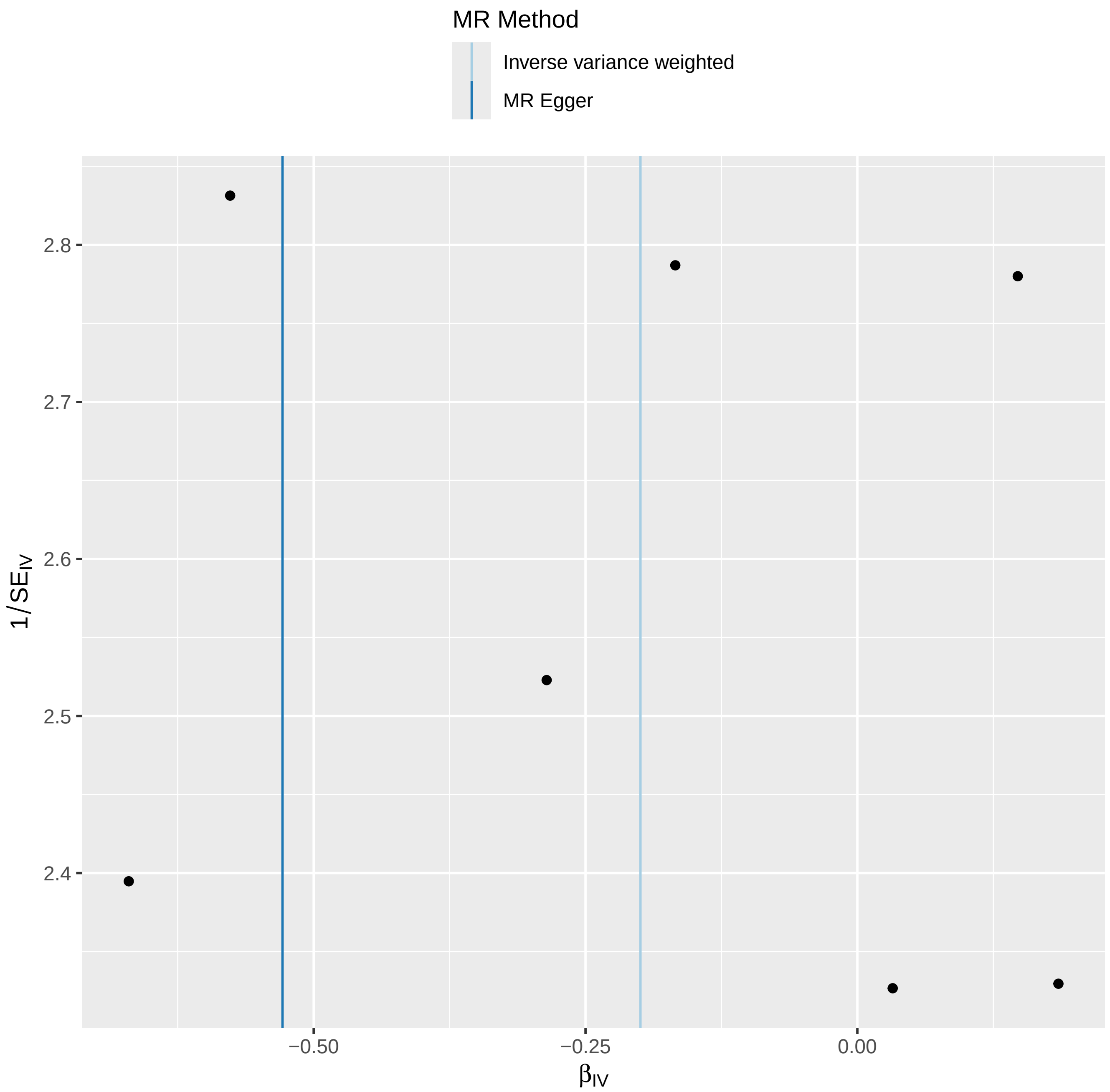

MR Method
Inverse variance weighted
MR Egger
2.8
2.7
2.6
2.5
2.4
1/SE_IV
−0.50
−0.25
0.00
β_IV
